# Prevalence and risk factors of obesity and hypertension among students at a central university in the West Bank

**DOI:** 10.3402/ljm.v7i0.19222

**Published:** 2012-10-15

**Authors:** Yasin I. Tayem, Nagham A. Yaseen, Wiam T. Khader, Lama O. Abu Rajab, Ahmad B. Ramahi, Mohammad H. Saleh

**Affiliations:** Department of Physiology and Pharmacology, Al-Quds University School of Medicine, Abu Dis, Jerusalem, West Bank, Palestinian Territory, Occupied

**Keywords:** obesity, hypertension, risk factors, young adults

## Abstract

**Objectives:**

We aimed to determine the prevalence and correlates of obesity and hypertension (HTN) among students at a central university in the West Bank.

**Materials and methods:**

This cross-sectional study targeted a cohort of 553 students (59.5% males, 40.5% females) aged 17–26 years (median = 21) from Al-Quds University. Body mass index (BMI) and blood pressure (BP) were measured. Participants completed a questionnaire on physical activity, sedentary behavior, dietary factors, smoking and family history of obesity, HTN, and coronary artery disease. The magnitude of correlation was assessed by Spearman's rho (*r*_s_) and Chi-square tests.

**Results:**

The prevalence of overweight was 25% (31.1% males, 15.6% females) and obesity 7.2% (9.4% males, 4% females). Obesity and overweight were associated with family history of obesity in both genders (*p*<0.001) and physical activity in males (*r*_s_= − 0.162, *p*<0.005). No correlation was demonstrated between participants’ BMI and sedentary lifestyle or consumption of fast food. Pre-HTN was detected in 27.1% (38% males, 11.2% females) and HTN in 2.2% (3.3% males, 0.4% females). Pre-HTN and HTN were associated with obesity (*r*_s_=0.252, *p*<0.001) and smoking (*p*<0.05). No relationship was detected between students’ BP and sedentary behavior, family history of HTN/CAD, or consumption of fast food. The prevalence of increased BMI and BP among males was significantly higher than females (*p*<0.001).

**Conclusions:**

We detected a high prevalence of elevated BP and excess weight gain among students at Al-Quds University. An interventional program is urgently needed to control these cardiovascular risk factors in this community.

Obesity often coexists with hypertension (HTN) and a linear relationship between blood pressure (BP) values and weight was observed (1). Wofford et al. reviewed the association between obesity and HTN and indicated that excess weight gain accounts for 65–75% of the risk for essential HTN (1).

Over the last few decades, obesity has become a global public health concern. In 2005, the estimated total number of obese and overweight adults around the globe was 396 million and 937 million, respectively (2). Although obesity has been widely reported in industrialized nations, in recent years, there has been an ever-increasing prevalence in developing countries. In Kuwait, for instance, the prevalence of obesity in 2008–2009 was around 77% for both genders (3). Many factors underlie this growing rate of excess weight gain in the developing world, but it is thought to result from significant lifestyle changes which have been evolving in these societies (4).

Middle Eastern countries, including Palestine, have witnessed major lifestyle transformations due to rapid urbanization, increasing sedentary behaviors, and proliferation of high-fat diet. These lifestyle changes have had a considerable impact on increasing obesity and reducing physical requirements of daily life (4). Consequently, these lifestyle changes are thought to be majorly responsible for the epidemic of non-communicable diseases in this region (5).

The prevalence of HTN in developing countries appears to be on the rise. In the Mediterranean region, HTN affects 26% of the total population (6). Martins et al. determined the proportion of pre-HTN and HTN among college students in Brazil. The authors reported that the prevalence of elevated BP was 9.7% and was higher among males. Moreover, they showed that the rate of excess weight was 18.2% and that the increase in body mass index (BMI) was associated with an elevation in mean BP (7). Because HTN is a significant, but modifiable, risk factor for cardiovascular diseases, strategies to achieve even a modest lowering of the levels of BP in the population should be an important public health goal.

An attempt was made in the present investigation to determine the prevalence of and correlation between HTN and obesity among students at a central university in the West Bank. Moreover, we aimed to determine risk factors of these conditions including smoking, lifestyle behaviors, dietary factors, and family history. An understanding of these risk factors in this community will help identify and control cardiovascular risk factors occurring at young age in Palestine.

## Materials and methods

### Ethics

Ethical approval was provided by the Al-Quds University Human Research Ethics Committee. Signed consent forms were sought from participants using letters containing detailed information about the purpose and nature of the study.

### Sampling size

The sample size from each college was proportional to the number of students attending that particular college. Students selected were from the 15 undergraduate colleges of Al-Quds University located in the West Bank.

### Sampling

This was a descriptive cross-sectional university-based study, which targeted a cohort of 553 randomly selected students (59.5% males, 40.5% females). All students aged 17–26 years enrolled at all Al-Quds University undergraduate programs were eligible to participate in this study.

### Data collection

Data on age, gender, physical activity, sedentary behavior, dietary habits, smoking (cigarettes or hookah) and family history of obesity, HTN, and coronary artery disease (CAD) were collected by interviewing participants. Physical activity measures included daily duration of walking and other sport activities. Participants were considered to have severe physical inactivity if their daily physical activity was <30 min/day. Sedentary lifestyle included daily time spent watching TV, surfing Internet, or playing video games. Subjects were categorized to have severe sedentary behavior if daily time spent on these activities was >121 min. Students who had at least one first-degree relative with obesity, HTN, or CAD were considered to have positive family history for these conditions. Concerning dietary factors students were considered to be high consumers of fast food or caffeinated drinks, if they consumed more than three fast food meals per week or greater than three cups of caffeine-containing drinks (coffee, tea, cola, and energy drinks) per day, respectively. Data were collected between March and June 2012.

### Outcome measurements

#### BP measurement

The measurement of BP was performed manually by trained personnel using a mercury sphygmomanometer after the subject had rested for 5 min in the sitting position having had no cigarettes, coffee, or tea. In the case that systolic BP was higher than 140 mmHg or diastolic BP was more than 90 mmHg, the subject was requested to rest for 5 min, another BP measurement was taken, and the average of the two measurements was calculated. BP was categorized based on the recommendations of the Seventh Report of the Joint National Committee of Prevention, Detection, Evaluation and Treatment of High BP (JNC VII). The classification of BP (expressed in mmHg) for adults aged 18 years or older was as follows (8): Normal if systolic BP was lower than 120 and diastolic BP was lower than 80, pre-HTN if systolic BP was 120–139 and/or diastolic BP was 80–89, and HTN if systolic BP was >140 and/or diastolic BP was > 90.

#### Anthropometric measurements

For the screening of overweight and obesity, with the subject standing and shoes and jacket removed, height was measured by Leicester Height Measure (to the nearest 0.005 m). Weight was measured (to the nearest 0.1 Kg) by using a digital scale (Tanita BC 587, Illinois, USA). BMI, defined as body weight in kilograms divided by the square of height in meters, was calculated as a measure of weight category. BMI (expressed in kg/m^2^) was categorized as follows: underweight <18.5; normal weight 18.5–24.9; overweight 25–29.9; and obese >30 (9).

## Statistical analysis

Spearman's Rank Non-Parametric Correlation Coefficient (*r*_s_) was quoted to reflect the magnitude of correlation between BP and BMI categories and these parameters with physical activity, sedentary behavior, and dietary factors. Chi-square test was utilized to determine whether BP/BMI categories were related to gender, smoking, and family history of HTN, CAD, and obesity. Data were analyzed using SPSS version 17.0. The statistical significance was set at *p*<0.05.

## Results

This study targeted a cohort of 553 students aged 17–26 years. The median age of participants was 21 years out of whom 25% of were below the age of 20 years and 75% were above 22 years. The gender distribution was 224 females (40.5%) and 329 males (59.5%).

As for the prevalence of associated factors of obesity and HTN examined in this study (Table 1), out of all participants examined, 29.3% were smokers (42.2% males, 10.3% females), 52.4% were high consumers of fast food (62.9% males, 37.1% females), 45.6% of students consumed a lot of caffeine-rich drinks (52% males, 35.6% females), 26.9% had severe physical inactivity (14.6% males, 45.1% females), and 57.7% had severe sedentary behavior (56.5% males, 58.9% females).

**Table 1:** The prevalence of risk factors of obesity and hypertension (HTN) among a central university students in the West Bank (*n =* 553; males = 329, females = 224)

	Males (%)	Females (%)	All (%)
Smoking			
Yes	139 (42.2)	23 (10.3)	162 (29.3)
No	190 (57.8)	201 (89.7)	391 (70.7)
Fast-food consumption			
None	46 (14)	46 (20.5)	92 (16.6)
1–2 meals/week	76 (23.1)	95 (42.4)	171 (30.9)
> 3 meals/week	207 (62.9)	83 (37.1)	290 (52.4)
Caffeine-rich drinks consumption			
None	28 (8.5)	37 (16.5)	65 (11.8)
1–2 cups/day	130 (39.5)	106 (47.3)	236 (42.7)
3–4 cups/day	171 (52)	81 (35.6)	252 (45.6)
Physical activity			
< 30 min/day	48 (14.6)	101 (45.1)	149 (26.9)
31–60 min/day	66 (20.1)	49 (21.9)	115 (20.8)
> 61 min/day	215 (65.3)	74 (33)	289 (52.3)
Sedentary lifestyle			
< 60 min/day	67 (20.4)	49 (21.9)	116 (21)
61–120 min/day	76 (23.1)	43 (19.2)	119 (21.5)
> 121 min/day	186 (56.5)	132 (58.9)	318 (57.7)

The prevalence of excessive weight gain (Table 2) and the magnitude of association between overweight/obesity and their risk factors were examined in this study (Table 3). Overweight was detected in 25% of participants (31.1% males, 15.6% females) and obesity in 7.2% (9.4% males, 4% females). Excess BMI was more common in males (*p*<0.001) and was associated with family history of obesity (*p*<0.001) in both genders and with physical activity in males (*r*_s_= − 0.162, *p*<0.01). No association was detected between anthropometric parameters and sedentary behavior, high consumption of fast food, and caffeinated drinks or family history of CAD.

**Table 2:** Blood pressure (BP) and weight status categorization according to gender among a central university students in the West Bank (*n*=553; males = 329, females = 224)

	Boys (%)	Girls (%)	All (%)
Underweight	7 (2.1)	13 (5.8)	20 (3.6)
Normal BMI	188 (57.1)	167 (74.6)	355 (64.2)
Overweight	103 (31.1)	35 (15.6)	138 (25)
Obese	31 (9.4)	9 (4)	40 (7.2)
Normotensive	193 (58.7)	198 (88.4)	391 (70.7)
Pre-hypertensive	125 (38)	25 (11.2)	150 (27.1)
Hypertensive	11 (3.3)	1 (0.4)	12 (2.2)

**Table 3:** Magnitude of correlation between obesity and hypertension (HTN) and their risk factors among students at a central university in the West Bank (*n*=553; males = 329, females = 224)

	BMI category	BP category
BMI category		0.252**
Physical activity	−0.029	0.12**
Sedentary lifestyle	−0.032	−0.026
Fast-food consumption	−0.031	0.046
Caffeinated drinks consumption	0.023	0.084*

Values represent Spearman's rho (*r*_s_).

* Correlation is statistically significant at 0.05 level (two-tailed).

** Correlation is statistically significant at 0.01 level (two-tailed).

The rate of elevated BP (Table 2) and its risk factors were also assessed in this investigation (Table 3). Pre-HTN19222was detected in 27.1% (38% males, 11.2% females) and HTN in 2.2% (3.3% males, 0.4% females). High BP was associated with excessive weight gain (*r*_s_=0.252, *p*<0.001) and smoking (*p*<0.05). Consumption of caffeinated drinks was weakly correlated with BP but did not reach statistical significance (*r*_s_=0.084, *p*=0.05). No association was detected between BP and sedentary behavior, family history of HTN/CAD, or consumption of fast food. Similar to obesity, the prevalence of HTN among males was significantly higher than females (*p*<0.001).

## Discussion

This study was carried out to examine the prevalence and risk factors of HTN and obesity among students at a central university in the West Bank. Our data demonstrated an alarming rate of increasing weight gain, raised BP, and dissemination of sedentary behavior among students. Results of this study emphasized the need for adopting a health policy to assess and control obesity and HTN and their risk factors at young age in Palestine.

Our findings demonstrated that a large proportion of students suffered from physical inactivity and sedentary behavior (especially girls). Smoking, consumption of unhealthy food and caffeinated drinks were also very common (especially boys). Al-Hazzaa et al. reported similar results among Saudi adolescents. The authors showed that screen time and inactive lifestyle were more common among females while males consumed more unhealthy diet (10). Our data and the reports of others emphasized that young adults in the Middle East are adopting a Westernized way of life and that they are likely to face difficulties related to lifestyle choices similar to those in the developed world.

The results of this study showed that a quarter of the students were overweight and 7.2% were obese. Abdeen et al. reported that the rate of overweight in Palestinian adults in 1999–2000 was 35.5% in women and 40.3% in men while obesity was detected in 31.5% in women and 17.5% in men. Similar to our findings, the authors showed that the rate of obesity was lower in women than men at young age (11). This may be related to the fact that as women get older they suffer from increasing lack of exercise, sedentary behavior, and wide availability of indoor food stimuli. Our findings and other investigators’ reports showed that obesity has become a public health concern in the developing world that needs urgent attention.

We also explored the risk factors of obesity and HTN among students. A strong correlation between physical inactivity and anthropometric parameters was noticed in males but no relationship with sedentary behavior or consumption of fast food was detected. Al-Hazzaa et al. showed that obesity was more common in subjects who were physically inactive but no association with screen time or unfavorable dietary habits was found (10). Family history of obesity was also found to be associated with obesity in this study. In agreement with this, Yücel et al. reported that paternal obesity and having an obese sibling were significant risk factors for excess BMI (12). These findings support the need to promote active living among young generations to control the increasing rate of obesity especially among those with positive family history.

The prevalence of high BP and its associated factors were also observed in this investigation. Our data demonstrated that around one quarter of the students had pre-HTN whereas HTN was evident in 2.2% with males being significantly more affected than females. Sawalha et al. conducted a study to determine risk factors in Palestinian patients with ischemic stroke and found that HTN was the most common modifiable risk factor for this disease (13). Notably, we found that the majority of students who suffered from high BP actually had pre-HTN. Similarly, Ortiz-Galeano et al. reported a high rate of pre-HTN in young adults in Spain (24%) (14). These data emphasize the importance of BP screening for young adults to detect any increase in BP at an early age.

Our data also demonstrated that the rate of high BP was associated with obesity and smoking. Wakabayashi et al. reported that adiposity was strongly associated with HTN (15). In contrast to our results that physical activity was not associated with BP, Dimeo et al. reported that aerobic exercise reduced BP in resistant HTN (16). We were not able to identify association between raised BP and fast-food consumption or screen time. Kim et al. reported that the vegetable-rich traditional Korean diet did not show any protective effect on HTN (17). In contrast to our findings, however, Tringler et al. found a close relationship between sedentary lifestyle and the development of high BP (18). Excessive consumption of caffeinated drinks was weakly associated with BP but this did not reach statistical significance. These findings demonstrate the importance of encouraging students to maintain active living, stop smoking, and control weight in order to avoid dangerous rises in BP.

## Conclusions

Students at Al-Quds University suffered from an increased prevalence of excessive weight gain and elevated BP. A high rate of smoking, sedentary behavior, physical inactivity, excessive consumption of unhealthy food, and caffeine-rich drinks was also revealed. Our findings emphasize the importance of promoting active lifestyle, smoking cessation, and screening for obesity and HTN among students to control cardiovascular risk factors in this community.
